# Effect of Rifampin on the Single-Dose Pharmacokinetics of Oral Cabotegravir in Healthy Subjects

**DOI:** 10.1128/AAC.00487-17

**Published:** 2017-09-22

**Authors:** S. L. Ford, K. Sutton, Y. Lou, Z. Zhang, A. Tenorio, C. Trezza, P. Patel, W. Spreen

**Affiliations:** a GlaxoSmithKline, Research Triangle Park, North Carolina, USA; b ViiV Healthcare, Research Triangle Park, North Carolina, USA; c PAREXEL International, Durham, North Carolina, USA

**Keywords:** drug-drug interactions, HIV-1, integrase strand transfer inhibitors, cabotegravir, rifampin, pharmacokinetics

## Abstract

Drug-drug interactions between antiretroviral medications and rifampin complicate the treatment of HIV and tuberculosis coinfection. This study evaluated the effect of rifampin on the pharmacokinetics of oral cabotegravir, an integrase strand transfer inhibitor being investigated for long-acting treatment and prevention of HIV-1 infection. This was a phase I, single-center, open-label, fixed-sequence crossover study in healthy adults. The objective was to evaluate the effect of steady-state rifampin on the single-dose plasma pharmacokinetics of cabotegravir. Subjects received a single oral dose of cabotegravir (30 mg) on day 1 followed by plasma sampling on days 1 to 8. Treatment with once-daily oral rifampin (600 mg) occurred on days 8 to 28. Subjects received a second dose of 30 mg cabotegravir on day 21 followed by pharmacokinetic sampling on days 21 to 28. Fifteen subjects were enrolled and completed the study. Rifampin decreased the cabotegravir area under the concentration-time curve from 0 h to infinity and the half-life by 59% and 57%, respectively, whereas oral clearance was increased 2.4-fold. The maximum concentration of cabotegravir in plasma was unaffected by coadministration with rifampin. All adverse events were mild in severity, with chromaturia attributed to rifampin observed in all subjects. Rifampin induction of cabotegravir metabolism resulted in increased cabotegravir oral clearance and significantly decreased cabotegravir exposures. Rifampin is expected to increase cabotegravir clearance following long-acting injectable administration. Concomitant administration of rifampin with oral and long-acting formulations of cabotegravir is not recommended currently without further study. (This study has been registered at ClinicalTrials.gov under registration no. NCT02411435.)

## INTRODUCTION

Coinfections of HIV-1 and tuberculosis (TB) are common globally, with at least one-third of individuals living with HIV also infected with Mycobacterium tuberculosis and 35% of all deaths in patients with HIV-1 infection in 2015 attributed to TB (1). Studies have demonstrated increased survival (2) and reduced risk of TB relapse (3) with early initiation of antiretroviral therapy (ART) for coinfected subjects, demonstrating a critical need for safe and effective treatment regimens for both infections. However, significant drug-drug interactions between many antiretroviral agents and antituberculosis drugs exist, particularly rifampin (RIF) (4), a first-line component of TB regimens (5).

Rifampin is a potent inducer of hepatic drug-metabolizing enzymes, including cytochrome P450s (CYPs) and uridine 5′-diphospho-glucuronosyltransferases (UGTs) (6). Concomitant treatment with RIF reduces the concentrations of most protease inhibitors (PIs), nonnucleoside reverse transcriptase inhibitors (NNRTIs), and integrase strand transfer inhibitors (INSTIs) used to treat HIV-1 through the induction of CYP3A4- and UGT-mediated metabolism (4) in plasma. Decreased plasma exposure of antiretroviral agents can lead to the emergence of antiretroviral resistance, virologic failure, and progression of HIV-1-related diseases (7, 8). Thus, TB treatment in patients taking ART necessitates considerations such as ART switching, dosing modifications (as is the case with INSTIs), or replacement of rifampin with rifabutin (RFB), another rifamycin that exerts a smaller inductive effect than that of RIF on enzymatic drug metabolism.

Cabotegravir (CAB) is an INSTI in development for the treatment and prevention of HIV-1 infection (9). Cabotegravir has been formulated as a long-acting injectable nanosuspension (CAB LA) with a favorable pharmacokinetic (PK) profile supporting monthly or bimonthly administration (10) and a potential to overcome barriers to adherence associated with daily oral ART and preexposure prophylaxis (PrEP). Induction of suppression of HIV infection has been achieved with oral CAB in combination with 2 NRTIs in antiretroviral-naive subjects (11), while maintenance of HIV suppression has been achieved with a 2-drug regimen of oral CAB (10 mg, 30 mg, or 60 mg) once daily (QD) with 25 mg rilpivirine (RPV) once daily, with geometric mean plasma Cτ (predose [trough] concentration at the end of the dosing interval) concentrations 8-, 24-, and 50-fold above the protein-adjusted 90% inhibitory concentration (PA-IC_90_) of 0.166 μg/ml (11). Monthly or bimonthly CAB LA + RPV LA has also demonstrated durable maintenance of viral suppression, with average concentrations at trough 8-fold above the PA-IC_90_ or higher (D. A. Margolis, D. Podzamczer, H.-J. Stellbrink, T. Lutz, J. B. Angel, G. Richmond, B. Clotet, F. Gutierrez, L. Sloan, S. K. Griffith, M. St Clair, D. Dorey, S. Ford, J. Mrus, H. Crauwels, K. Y. Smith, P. E. Williams, and W. R. Spreen, presented at the 21st International AIDS Conference, 18 to 22 July 2016, Durban, South Africa). Cabotegravir LA is also being developed as a single agent to be used for PrEP (9). Although efficacy has been demonstrated at CAB doses of 10 mg (CAB 10 mg) and higher, CAB 30 mg has been selected as the oral lead-in dose as a safety check prior to initiating CAB LA injections that achieve concentrations above the 10-mg dose. Oral CAB 30 mg is administered QD with a terminal elimination phase half-life (*t*_1/2_) of 40 h (9). Cabotegravir is metabolized primarily through UGT1A1 with a minor contribution of UGT1A9 (11); therefore, RIF has the potential to reduce CAB concentrations in plasma with concurrent administration, resulting in subsequent loss of efficacy. This study was undertaken to determine the effect of steady-state RIF on single-dose oral CAB PK in healthy adults in order to inform use of RIF with CAB LA.

## RESULTS

### Subjects.

Fifteen subjects were enrolled in the study, and all completed it as planned. The majority of subjects were male (67%) and white (80%), with a mean age of 48.5 years (range, 21 to 65 years) (Table 1).

**TABLE 1:** Baseline demographics^*a*^

Characteristic, unit	All subjects (*n* = 15)
Age, yr	48.5 (14.1)
Male, *n* (%)	10 (67)
BMI,^*b*^ kg/m^2^	26.7 (3.6)
Height, cm	172.4 (7.0)
Weight, kg	79.6 (12.8)
Ethnicity, *n* (%)	
Hispanic	1 (7)
Not Hispanic or Latino	14 (93)
Race, *n* (%)	
Black	3 (20)
White	12 (80)

a Unless otherwise specified, values are means (SD).

b BMI, body mass index.

### Pharmacokinetic analyses.

**FIG 1:**
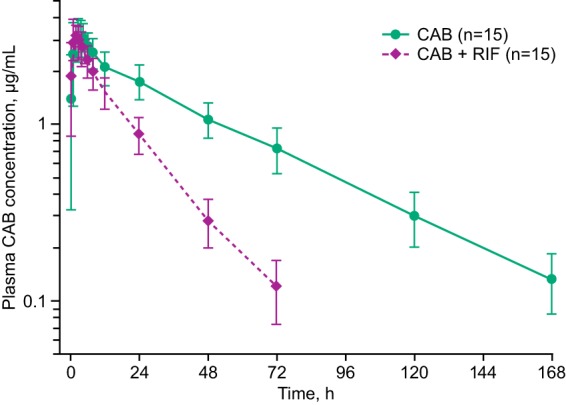
CAB concentration in plasma-time profiles following administration with and without RIF. Concentration values are means ± SD. CAB, cabotegravir; RIF, rifampin.

Single-dose plasma CAB exposures were reduced when CAB was administered concomitantly with RIF. A summary of CAB PK parameters and statistical comparisons is presented in Table 2. Figure 1 shows the mean CAB concentration in plasma versus time profiles for CAB administered alone, for which CAB concentrations were quantifiable in all subjects through 168 h of sampling, and for CAB administered with RIF, for which the mean concentration in plasma fell below the limit of detection of the assay after the 72-h sample. Coadministration of RIF 600 mg once daily with single-dose CAB 30 mg increased apparent oral clearance (CL/F) 2.4-fold (90% confidence interval [CI], 2.2 to 2.8) and decreased CAB area under the concentration-time curve from 0 h to infinity (AUC_0–∞_) by 59% (90% CI, 54% to 64%) and *t*_1/2_ by 57% (90% CI, 54% to 61%) compared with CAB 30 mg alone. The maximum observed concentration of cabotegravir in plasma (*C*_max_) was unaffected by RIF coadministration.

**TABLE 2:** Summary of plasma CAB PK parameters and treatment comparisons^*a*^

Parameter	Geometric mean (95% CI)		GLSM ratio (90% CI)
	CAB^*b*^ (*n* = 15)	CAB + RIF^*c*^ (*n* = 15)	CAB + RIF:CAB
AUC_0–∞_, μg · h/ml	146 (128–167)	59.7 (52.8–67.5)	0.41 (0.36–0.46)
*C*_max_, μg/ml	3.61 (3.28–3.96)	3.39 (3.05–3.76)	0.94 (0.87–1.02)
CL/F, liters/h	0.205 (0.180–0.234)	0.502 (0.444–0.568)	2.4 (2.2–2.8)
*t*_1/2_, h	38.5 (35.7–41.6)	16.4 (14.7–18.2)	0.43 (0.39–0.46)

a Abbreviations: AUC_0–∞_, area under the concentration-time curve from 0 h to infinity; CAB, cabotegravir; CI, confidence interval; CL/F, apparent clearance; *C*_max_, maximum observed concentration; GLSM, geometric least-squares mean; PK, pharmacokinetics; RIF, rifampin; *t*_1/2_, terminal elimination phase half-life.

b Following a single dose of CAB 30 mg on day 1.

c Following a single dose of CAB 30 mg on day 21 with once-daily administration of 600 mg RIF (days 8 to 28).

### Safety.

All 15 subjects reported at least 1 adverse event (AE) during the course of the study, the most frequent of which was chromaturia, a recognized adverse effect of RIF that occurred in all 15 subjects (100%) during dosing with RIF 600 mg on days 8 to 28. Other AEs considered related to study drugs by the investigator were headache (*n* = 1; 7%) after the initial CAB dose and decreased appetite (*n* = 2; 13%) and fatigue (*n* = 1; 7%) during days 8 to 20. Adverse events considered not related to study medications included dyspepsia, nausea, cellulitis, erythema migrans, second-degree burn, pruritus, and hematoma (*n* = 1; 7% for each). All AEs were grade 1 (mild) in severity. No clinically significant trends were observed in laboratory values, electrocardiograms (ECGs), or vital signs. No deaths or serious AEs were reported, and no subjects were withdrawn from the study.

## DISCUSSION

Steady-state RIF increased CAB oral clearance 2.4-fold, which resulted in a decrease in plasma CAB AUC_0–∞_ of 59% and in *t*_1/2_ of 57% following a single oral dose of CAB 30 mg. Cabotegravir *C*_max_ was unaffected by RIF coadministration, which is consistent with RIF-induced metabolism via enhanced UGT1A1 metabolism rather than changes to oral bioavailability. The reduction in CAB exposure is consistent with reduction in exposure to the INSTIs dolutegravir (DTG) and raltegravir (RAL) when coadministered with RIF. Dolutegravir is metabolized primarily through UGT1A1, with a minor contribution from CYP3A (12). Rifampin decreased the AUC_0–∞_ of once-daily DTG by 54% and the minimum observed concentration in plasma (*C*_min_) by 72% compared with DTG alone (13). Similarly, RIF reduced the plasma exposure of RAL, which is subject to metabolism via UGT1A1, by 40% and *C*_min_ by 61% (14). Interactions between RIF and DTG or RAL can be overcome by increasing the dosing frequency or dosage, specifically by increasing DTG from 50 mg once daily to 50 mg twice daily and increasing RAL from 400 mg twice daily to 800 mg twice daily.

Although it is expected that CAB 30 mg once daily when coadministered with RIF will maintain steady-state Cτ above 8 times the PA-IC_90_, approximately equivalent to the 10-mg oral dose that demonstrated efficacy in maintaining suppression of HIV (10), it no longer achieves concentrations consistent with its intended use as a safety check for CAB LA exposures above this range when induction of UGT is no longer evident. Doubling the dose or increasing the dosing frequency of oral CAB when coadministered with RIF may mitigate the interaction observed in this study; however, this interaction is also expected to affect the PK profile following CAB LA, in which dose modification in the clinical setting would present a challenge due to injection volume constraints and/or increased dose frequency. Additional studies would be required to evaluate the feasibility of dose modification of either oral CAB or CAB LA. The potential for coadministration of CAB and RFB, another antimycobacterial agent with less induction potential, will be evaluated in a future study. Furthermore, in current phase IIb/III trials of HIV-infected subjects, CAB LA is coadministered with RPV LA. Rifampin decreases exposure to RPV following oral administration (15), and RPV coadministration with RIF is contraindicated (16). Subjects who require RIF treatments are excluded from participation in clinical trials of CAB LA + RPV LA (17, 18).

Cabotegravir and RIF, alone and in combination, were well tolerated in this study. All AEs reported by subjects were considered mild in severity. The most common drug-related effect was chromaturia, a well-documented and benign side effect of RIF (19), which occurred in all subjects. No deaths, serious AEs, or clinically significant laboratory abnormalities occurred during the study.

In conclusion, coadministration of RIF with oral CAB 30 mg once daily is not recommended. Rifampin should not be coadministered with CAB LA + RPV LA in HIV-infected patients. Additional studies are needed to address alternative approaches to treating TB infection in patients receiving CAB, including using RFB, switching to other ART regimens, or modifying the dose amount or administration of CAB or CAB LA.

## MATERIALS AND METHODS

### Study design and subject demographics.

This was a phase I, single-center, open-label, fixed-sequence crossover study in healthy adults to evaluate the effect of steady-state RIF on the PK and safety of oral CAB 30 mg. Healthy men and women between 18 and 65 years of age with body weight of ≥50 kg and body mass index of 18.5 to 31.0 kg/m^2^ were eligible for the study. During the trial, women of childbearing potential were required to be sexually inactive by abstinence or to use contraceptive methods with a failure rate of <1% (with the exception of oral contraceptives because of their interaction with rifampin). Subjects were required to have negative screening results for HIV-1, hepatitis B, and hepatitis C. Subjects were excluded if they had a current or chronic history of liver disease, known hepatic or biliary abnormalities, or a history of clinically significant cardiovascular disease. Subjects were asked to abstain from taking prescription and nonprescription drugs, including vitamins and herbal products, within 7 days of the first dose of study medication until completion of the follow-up visit. Subjects had a screening visit within 30 days prior to the first dose of the study drug and a follow-up visit within 10 to 14 days of the last dose of the study drug in addition to multiple visits while being treated. Written informed consent was obtained from each subject prior to the performance of any study-specific procedure.

This study was conducted in accordance with the International Conference on Harmonisation of Technical Requirements for Registration of Pharmaceuticals for Human Use, Good Clinical Practice, all applicable patient privacy requirements, and the ethical principles outlined in the Declaration of Helsinki (2013). The study protocol and informed consent document were reviewed and approved by a regional institutional review board (Midlands Institutional Review Board, Overland Park, KS, USA). The study was registered with ClinicalTrials.gov (NCT02411435).

### Objectives.

The primary objective of this study was to compare the PK of single-dose oral CAB 30 mg when coadministered with RIF 600 mg once daily at steady state to that of CAB alone. The primary endpoints were the CAB *C*_max_ and AUC_0–∞_. Secondary endpoints included CL/F, *t*_1/2_, AEs, clinical laboratory evaluations, ECGs, and vital sign assessments.

### Procedures.

**FIG 2:**
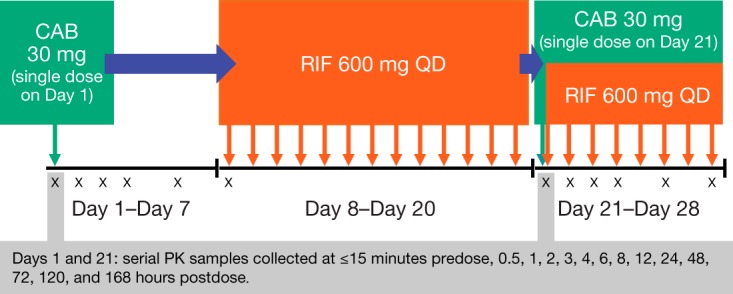
Study design. All subjects received the same study medication throughout the trial. Both CAB 30 mg and RIF 600 mg were administered to subjects on day 21. CAB, cabotegravir; QD, once daily; RIF, rifampin.

On day 1, a single dose of CAB 30 mg was administered to each subject under fasting conditions (Fig. 2). Blood samples to determine PK concentrations were obtained ≤15 min prior to dosing and at 0.5, 1, 2, 3, 4, 6, 8, 12, 24, 48, 72, 120, and 168 h postdose to fully characterize AUC_0–∞_ and *t*_1/2_. Following safety assessments and PK sampling on day 8, subjects began oral dosing of RIF 600 mg (two 300-mg capsules) once daily for 13 days with a full glass of water (240 ml) either 1 h before or 2 h after a meal at the same time each day. On day 21, subjects received another single dose of CAB 30 mg and continued RIF 600 mg for 7 additional days through the completion of serial PK sampling.

Safety assessments included monitoring of AEs, clinical laboratory tests, vital signs, and ECGs. Physical examinations and pregnancy tests were also conducted. Study staff were responsible for detecting, documenting, and reporting events that met the definition of an AE; AE information volunteered by the subject or detected by other means was collected from the start of study treatment until the follow-up visit.

### Bioanalytical methods.

Blood samples were taken via an indwelling cannula (or by direct venipuncture), collected into a 3-ml tripotassium-EDTA (K_3_EDTA)-treated tube, and immediately cooled on ice. Within 1 h of collection, the plasma was separated by refrigerated (4°C) centrifugation at 1,500 to 2,000 × *g* for a minimum of 10 min. Supernatant plasma was transferred into a single 1.8-ml cryovial and kept frozen at −20°C or on dry ice until measurement. Following protein precipitation, the sample supernatant was diluted and analyzed for CAB using a validated liquid chromatography/mass spectrometry assay. The range of quantification for CAB using a 25-μl aliquot of K_3_EDTA-containing plasma was 25 to 25,000 ng/ml. Quality control samples containing CAB at 3 known concentrations were stored and analyzed with each batch of samples against separately prepared calibration standards. For an analysis to be acceptable, no more than one-third of the quality control results were to deviate from the nominal concentration by more than 15%, with ≥50% of the results from each sample within 15% of the nominal value.

### Statistical methods.

A sample size of 15 was required to obtain 12 evaluable subjects, accounting for a predicted withdrawal rate of 20% and the within-subject variability of CAB. The within-subject variations in PK parameters from previous oral CAB studies were in the range of 6.7% to 7.8%, and the CAB PK parameters AUC_0–*t*_, AUC_0–∞_, and *C*_max_ were 6.9% to 11.4% (data not shown). Based on a within-subject coefficient of variation of 11.4% and a sample size of 12, the lower and upper bounds of the 90% CI for the treatment difference on a logarithmic scale were estimated to occur within 8.3% of the point estimate for AUC_0–*t*_, AUC_0–∞_, and *C*_max_.

CAB concentration in plasma-time data were analyzed by noncompartmental methods based on the actual sampling times with WinNonlin Phoenix 6.3 (Certara Corporation, Princeton, NJ). Plasma PK parameters, which were calculated from the concentration in plasma-time data, included the following: AUC_0–∞_, AUC_0–*t*_, *C*_24h_, *C*_max_, time of *C*_max_, *t*_1/2_, and CL/F. The point estimates of the geometric least-squares mean ratio for the PK parameters (test/reference) and the associated 90% CIs were provided for treatment comparisons. The PK parameters were log transformed before analysis, and treatment comparisons were expressed as ratios on the original scale. Safety data were tabulated and summarized descriptively.
